# Existing eHealth Solutions for Older Adults Living With Neurocognitive Disorders (Mild and Major) or Dementia and Their Informal Caregivers: Protocol for an Environmental Scan

**DOI:** 10.2196/41015

**Published:** 2022-11-04

**Authors:** Ambily Jose, Maxime Sasseville, Samantha Dequanter, Ellen Gorus, Anik Giguère, Anne Bourbonnais, Samira Abbasgholizadeh Rahimi, Ronald Buyl, Marie-Pierre Gagnon

**Affiliations:** 1 Department of Family and Emergency Medicine Faculty of Medicine Université Laval Quebec City, QC Canada; 2 Faculty of Nursing Sciences Universite Laval Quebec City, QC Canada; 3 Vitam-Research Center in Sustainable Health Centre Intégré de Santé et de Services Sociaux de la Capitale-Nationale Quebec, QC Canada; 4 Department of Biostatistics and Medical Informatics Faculty of Medicine and Pharmacy Vrije Universiteit Brussels Brussels Belgium; 5 Frailty in Ageing Research Group Department of Gerontology Vrije Universiteit Brussel Brussels Belgium; 6 Geriatrics Department Universitair Ziekenhuis Brussel Brussels Belgium; 7 Faculty of Nursing Université de Montréal Montreal, QC Canada; 8 Department of Family Medicine McGill University Montreal, QC Canada; 9 Mila-Quebec AI Institute Montreal, QC Canada

**Keywords:** dementia, eHealth solutions, mild cognitive impairment (MCI), environmental scan, digital health

## Abstract

**Background:**

Dementia is one of the main public health priorities for current and future societies worldwide. Over the past years, eHealth solutions have added numerous promising solutions to enhance the health and wellness of people living with dementia-related cognitive problems and their primary caregivers. Previous studies have shown that an environmental scan identifies the knowledge-to-action gap meaningfully. This paper presents the protocol of an environmental scan to monitor the currently available eHealth solutions targeting dementia and other neurocognitive disorders against selected attributes.

**Objective:**

This study aims to identify the characteristics of currently available eHealth solutions recommended for older adults with cognitive problems and their informal caregivers. To inform the recommendations regarding eHealth solutions for these people, it is important to obtain a comprehensive view of currently available technologies and document their outcomes and conditions of success.

**Methods:**

We will perform an environmental scan of available eHealth solutions for older adults with cognitive impairment or dementia and their informal caregivers. Potential solutions will be initially identified from a previous systematic review. We will also conduct targeted searches for gray literature on Google and specialized websites covering the regions of Canada and Europe. Technological tools will be scanned based on a preformatted extraction grid. The relevance and efficiency based on the selected attributes will be assessed.

**Results:**

We will prioritize relevant solutions based on the needs and preferences identified from a qualitative study among older adults with cognitive impairment or dementia and their informal caregivers.

**Conclusions:**

This environmental scan will identify eHealth solutions that are currently available and scientifically appraised for older adults with cognitive impairment or dementia and their informal caregivers. This knowledge will inform the development of a decision support tool to assist older adults and their informal caregivers in their search for adequate eHealth solutions according to their needs and preferences based on trustable information.

**International Registered Report Identifier (IRRID):**

DERR1-10.2196/41015

## Introduction

### Dementia is a Public Health Challenge

Neurocognitive disorders (NCDs), both major and minor, were generally termed dementia in many kinds of literature [1]. Hence, in this paper, the term dementia is used generally to represent all these conditions. Dementia is an important public health challenge in current and future societies worldwide. In 2021, the World Health Organization declared dementia a public health priority [2]. Dementia is a syndrome where the decline in cognitive function occurs beyond what might be expected from the usual consequences of organic aging. Alzheimer disease is the most common form of dementia [2,3]. Currently, more than 55 million people live with dementia worldwide, and its incidence is almost 10 million people each year. The prevalence is expected to increase to 70 million by 2030 and 139 million by 2050 [2]. Impacts on well-being also extend to informal caregivers of persons with dementia [4]. Few studies reported higher levels of depression, emotional distress, and physical strain in caregivers of persons with dementia than in caregivers of older adults with physical impairments [5,6].

### eHealth for Dementia Care

eHealth is defined as tools or treatments, mostly behavioral-based health interventions, delivered or enhanced through the internet, mobile devices, electronic/digital processes in communication, and related technologies [7]. eHealth implementation in dementia primarily focuses on improving the autonomy of persons with dementia [2]. eHealth assists primary caregivers by providing dementia-related knowledge and assistance to reduce their anxiety and depression, and thus, it can improve the health and well-being of both older people living with dementia and their informal caregivers [4].

Technological initiatives are undertaken at the national and regional level in Europe, Canada, and other countries to help people with dementia and their caregivers [8-10]. Effective eHealth interventions use a “blended” approach, combining remote support with direct coaching and interventions [4]. Some solutions are behavior based, with the primary focus on the self-efficacy of persons with dementia and their informal caregivers, and minimize depression and anxiety in caregivers [8,11,12].

Several literature reviews about eHealth solutions for older adults with dementia show that they are a promising strategy for enhancing the cognitive function of older people [11,13]. eHealth technology helps primary caregivers to assess and apply information related to dementia and offer better care for persons with dementia [14]. Many web-based solutions differ from each other in terms of objectives, features, functions, and specific subpopulations. For instance, the effectiveness of computerized cognitive training to delay the progression of cognitive impairment in people with mild and major NCD and dementia has been demonstrated [11]. Such solutions contain numerous exercises for different cognitive functions for people with mild cognitive impairment (MCI) and dementia, and they are more accessible and cost-effective in comparison to traditional cognitive interventions [11].

A systematic review on the effectiveness of internet-based interventions such as web-based self-management courses for dementia and apps to provide emotional support for family and caregivers shows a positive impact on caregivers’ stress reduction through regular web-based contact with health professionals by offering psychological support and necessary information on persons with dementia [5]. Another recent study emphasized that most of the informal caregivers of persons with dementia are interested in technology-based solutions, as most of them are younger than 65 years old and are familiar with computers in their workplace [15].

At the health care system level, there are various benefits to the implementation of effective eHealth solutions. It improves access to services in remote areas, service efficiency, and costs [4,14,16]. However, more work and effort are essential to fully achieve the potential of eHealth technologies for dementia care [10,14]. Hence, dementia-specific organizations such as the Alzheimer’s Society; local, national, or regional decision makers; and international organizations such as the World Health Organization should support the use of effective eHealth solutions.

One of the major obstacles to the large-scale implementation of effective eHealth solutions is scientific underreporting [5]. Most eHealth solutions are developed and tested over a short period of time, using only pilot evaluation approaches with limited samples or without a formal research/evaluation component [6]. Therefore, effective and user-friendly eHealth solutions that could be implemented on a large scale are difficult to identify. Additionally, variations in cognitive, sensory, and motor skills of persons with dementia in relation to technological advancement make it difficult to successfully assess effectiveness [6]. Research that supports informal caregivers of persons with dementia has shown that less than 3% of evidence-based interventions are effectively implemented into practice [5].

During the pandemic, eHealth was used to provide services such as web-based psychoeducation, self-management, and consultations to persons with dementia and their informal caregivers in many countries. A study from the Netherlands supports this finding. A Dutch survey also stated that video chatting and WhatsApp messaging were highly useful [4]. Similarly, the findings from a Taiwanese study also support the positive effect of telemedicine interventions on home-dwelling persons with dementia or MCI, as the telehealth intervention significantly reduced the participants’ gravity of neuropsychiatric symptoms and their primary caregivers’ stress levels [17]. Therefore, it is reasonable to say that now, more than ever, there is a need to provide eHealth support to persons with dementia and their informal caregivers [18].

As a part of the PROMISE project, a collaborative research initiative between Quebec (Canada) and Flanders (Belgium), we aim to identify promising eHealth solutions for persons with dementia and their informal caregivers. First, a systematic review of eHealth interventions was conducted for persons with dementia and their caregivers that is published elsewhere to obtain an overview of the existing body of knowledge about eHealth solutions for older adults with mild and major NCD and their informal caregivers [13]. These solutions include computer programs and web platforms to assist in personal organization, medication management, and household activities [18]. In fact, persons with dementia require additional solutions for location and navigation support, as there is a risk of wandering behavior and safety concerns. In addition, solutions offering leisure and reminiscence in persons with dementia were almost completely lacking.

The findings from our previous systematic review do not provide a complete portrayal of the available eHealth solutions for older adults with dementia or mild and major NCD and their informal caregivers. Therefore, we will conduct an environmental scan to gather comprehensive and up-to-date information on potential eHealth solutions that could be recommended to these people. An environmental scan is an efficient and organized means to collect relevant information regarding a new technology, as it is recognized as a good mechanism while expecting a change and improvement. Decision makers often use environmental scans to collect, organize, and analyze data. Moreover, an environmental scan was used to address the self-management of chronic diseases such as NCD [15]. When an environmental scan is properly executed, a series of evidence-based responses can be elicited from this method [12].

### Objectives

The purpose of this environmental scan is to identify eHealth solutions for older adults with dementia or mild and major NCD and their informal caregivers and to document their characteristics to inform the implementation of such solutions in Europe and Canada. The specific objectives are to

- inventory eHealth solutions for the targeted populations available in Europe and Canada and
- summarize the characteristics of these eHealth solutions, including their results and outcomes, implementation factors, and conditions of success.

## Methods

### Ethics Approval

The PROMISE project has been reviewed and approved by the ethics boards of the Centre Intégré de Santé et de Services Sociaux de la Capitale-Nationale (ref: MP-13-2019-1522) and Vrije Universiteit Brussel and Universitair Ziekenhuis Brussel (BUN 143201835242).

### Study Design

To achieve these objectives, we will perform an environmental scan. Although environmental scans are gaining popularity in the health sector and in research as a methodological approach to examine a specific health issue, there is no gold standard for this method [19]. The environmental scan is considered an effective assessment and data collection tool to analyze multifaceted issues, explore a policy, and critique articles. An environmental scan identifies the knowledge-to-action gap meaningfully [20]. Several studies have described the usefulness of environmental scans for assessing community needs for program and policy development [19].

As the environmental scan is adopted as an assessment tool in various contexts, it does not have a consistent definition. However, a working definition with details is essential to achieve the desired outcome [19]. It includes several steps from development to dissemination [20]. First, a team member should take the coordinator role. Second, stating the environmental scan purpose helps to keep it focused with a clear scope, and then imposing a timeframe helps to speed up the process. Brainstorming to determine all relevant resources and topics is essential. As the environmental scan progresses, it is important to involve identified stakeholders as needed. Critical analysis and synthesis of results help to make a summary report. Finally, the results and conclusions are shared with key stakeholders [20]. A similar methodology was followed to identify the extent and breadth of existing literature on older people’s perspectives on digital engagement and summarize the barriers and facilitators for technological nonuse, initial adoption, and sustained digital technology engagement [13].

### Search Strategy and Timeline

To complement the eHealth technologies identified previously in the systematic review [13], we will perform comprehensive bibliographic searches to identify recent eHealth solutions for older people with mild and major NCD or dementia and their informal caregivers. The initial data searches done by Dequanter et al [13] will be updated by two research assistants (AJ and SD) by gathering all available eHealth solutions in their respective jurisdictions (Canada and Europe) through databases and web searches. Identified solutions will then be reviewed by experienced investigators (MPG, MS, and RB).

The search strategy will include combinations of relevant keywords and their declinations, as presented in Textbox 1, and will be run in Google and search engines of websites.

**List of terms for the search strategies.**

**Terms dealing with the targeted population**

- Elderly, older people, caregiver, family caregiver, family caregiving, informal care, informal caregivers, aged, care partners

**Medical Subject Headings (MeSH) terms**

- Mild cognitive impairment, mild neurocognitive disorder, dementia, Alzheimer’s disease, neurodegenerative disorder, neurocognitive disorder, memory support, memory assist, memory help, cognition support
- Cognitive decline
- Cognitive disorder
- Cognitive dysfunction
- Cognitive impairment
- Frontotemporal
- Lewy Body
- Neurocognitive decline
- Neurocognitive dysfunction
- Neurocognitive impairment
- Vascular dementia

**Terms dealing with eHealth solutions**

- Information and communication technology services, eHealth, medical informatics, health informatics, mobile health, mHealth, telemedicine, telehealth, telecare, mobile devices, mobile applications, self-help applications, self-help devices, self-management applications, handheld computers, tablets, mobile phones, smartphones, personal digital assistant, mobile technology, health care robotics, assistive technology
- Intelligent systems, networked technology, telemonitoring, ambient-assisted living, active and assisted living, e-learning, activities of daily living, technologies, and virtual reality

**Additional terms**

- Prevention, prevent, adoption, use, nonuse, acceptance, community dwelling, community-based, nursing homes, day care centers, gerontechnology, psychoeducation, prescription

### Data Sources

We will also conduct targeted searches on relevant websites, including local, national, and regional organizations; private and public sector; and funding agencies. Three main sources will be searched for eHealth solutions for older adults with minor and major MCI or dementia:

1. General search: Academic sources will include Google Scholar, PubMed, and the Cochrane Library. The relevant keywords will be searched via Google and social networks (eg, Twitter and Instagram). We will consult the 10 first pages of results.
2. Targeted searches: The same search will be performed for each of the organizations listed in Textbox 2.
3. Expert consultation: For all ongoing eHealth projects in the field of dementia identified through websites and conference proceedings, we will contact the project lead if needed to complement the information.

**List of relevant organizations for the environmental scan.**

**Government agencies**

- Canadian Frailty Network, Public Health Agency of Canada, AgeWell, European Commission projects funded through the EU, Canadian Consortium on Neurodegeneration in Aging

**Public and private organizations**

- Digital Alzheimer Center of the Vrije Universiteit, Medical Center Amsterdam, Cordis Europa, Digital single market, Canadian institutes of health research, Active and Assistive Living programs, H2020 programs, Alzheimer Europe

**Foundations**

- Advocacy organizations patients/users (Dementia Alliance International, etc)

**Nongovernmental organizations**

- Research centers (Centre for Aging Brain innovations, etc)

**Practitioners and public health-related organizations**

- Industries
- Rural Dementia Action Research, Canadian Medical Association

**Patient and caregivers’ associations**

- Alzheimer Society Canada, Alzheimer Disease International, Women’s Brain Health Initiative

**Other relevant sites**

- Canadian Geriatrics Society, Canadian Academy of Geriatric Psychiatry

### Inclusion and Exclusion Criteria

The eHealth solutions must meet the following criteria: has the purpose to support or improve health and well-being in the daily life of the targeted population such as apps providing information about health or services, mental exercises and games, virtual assessments, and so forth; are produced within the last 5 years (so as of January 2018 up to the date of the search in 2022) in Europe and Canada; and must be available in these regions. We will include relevant solutions by any public or private entity, provided free of charge, or requiring some payment.

The eHealth solutions that are not currently available or for which we cannot confirm availability will be excluded. We will also discard any solution based on phone calls or one-way signals (ie, panic button), as they are not considered eHealth. Solutions that are not available in English, French, or Dutch will also be excluded.

### Data Extraction

We will use an extraction grid to document the characteristics of the technological solutions, based on the main attributes identified through the qualitative component of the larger research project. These attributes include the solution name, geographic availability, scientific evidence of impacts and evidence details, purpose, software technology, target population, domain, features, availability, a summary of the invention, targeted population (digital literacy level, cognitive/physical limitations, and ease of use), battery autonomy, design, compatibility, affordability, relevancy, URL, status/remarks, primary author, or company contact information (email and phone number). We will pilot the extraction grid on a sample of 5 solutions. One author (AJ) will then do the extraction for all identified technologies, and another author (MS or MPG) will check for accuracy.

### Analysis

We will gather detailed information based on the selected attributes and list all the available eHealth solutions for the targeted populations in Europe and Canada. This will provide data for the first objective. The next task is to analyze each technology based on the selected attributes, synthesize quantitative and qualitative data, and triangulate the results to understand implementation factors and conditions of success. We will use a narrative approach with charts and figures to summarize the results according to the key characteristics of the technological solutions. Classifying eHealth solutions based on their main function reveals their importance for patients and informal caregivers, along with their documented advantages, implications, and potential drawbacks. We will then prioritize the identified solutions according to the specific needs, expectations, and concerns of older adults with mild and major NCD or dementia and their informal caregivers. This prioritization will be informed by the qualitative component of the PROMISE project that consisted of consultations with older people with minor and major MCI and dyads of persons with dementia and their informal caregivers, and health and social care professionals [13]. These findings indicate that the most important attributes of eHealth solutions are perceived benefits (eg, well-being, autonomy, and self-confidence), risks (eg, burden and loss of autonomy), acceptability, feasibility, and costs.

## Results

The results of the environmental scan will be shared with all stakeholders who participate in this project. We will summarize the best available scientific evidence on each selected eHealth intervention in a brief plain language report for diffusion to a large audience. In Quebec, the team members will contribute to knowledge translation and mobilization through their involvement in important networks such as the Quebec Learning Health System Support Unit [21]. Quebec researchers will work closely with knowledge users and decision makers to raise awareness about the potential of eHealth for people with mild and major NCD or dementia, their informal caregivers, and health and social care providers. This can be achieved through ongoing collaborations with health and social care organizations, patient and informal caregiver associations, and national networks. In Flanders, the Flanders Expertise Centre on Dementia will offer active participation and facilitate contacts in their network of informal caregivers, persons with dementia, and relevant stakeholders. The Flanders Expertise Centre on Dementia will facilitate the implementation of the project’s results and support tools in their communication and training tools, and share them through their national and international network to promote uptake of the project’s output and sustain long-term effects and make them further transferrable by reaching the key stakeholders in daily care practice.

## Discussion

### Anticipated Findings

This environmental scan will provide timely knowledge about promising eHealth solutions for older adults with mild and major cognitive impairment or dementia and their caregivers. eHealth technology has changed the way people live in and out of their homes, and this revolution continues to make profound changes in better cognitive functions, activities of daily living, and safety [10,11]. Environmental scanning is an assessment method commonly used in business, quality improvement projects, and strategic planning projects and now is gaining popularity in the health sector and in research [18]. Although traditional public health principles differ from an environmental scan, it can lead to evidence-based findings [20]. For instance, the Centers for Disease Control and Prevention used an environmental scan to gather relevant information and share its results [19]. In this study, we expect to obtain pieces of evidence that support the best eHealth technologies.

One of the main limitations of the environmental scan method is its lack of consistent definition. The steps we follow in this study cannot be generalized in other circumstances. For example, in an organization, the environmental scan can be used to identify barriers and facilitators [19]. In the field of research, an environmental scan can be done for a scoping review [20], but in our study, it is done to gather evidence to support the development of a decision support tool. Based on the results of this environmental scan of eHealth solutions, the next phase of the project consists of developing a web-based decision support tool for persons with dementia and their informal caregivers. The purpose of the tool will be to facilitate informed decision-making regarding the choice of eHealth solutions that could be used for different purposes, such as dementia prevention, information and education, management and care, and support. In some studies, an environmental scan is the primary methodological approach. However, in other studies, an environmental scan is represented as one of the multiple methods used [19]. In this study, the environmental scan is considered in this way.

### Conclusion

There is an exponential number of initiatives undertaken to provide a better quality of life for older adults with minor and major NCD or dementia and their primary caregivers by using eHealth technologies. However, there is still much work to be done in optimizing research designs and methods. This environmental scan will provide insights into the characteristics of eHealth solutions that are beneficial for persons with dementia and their informal caregivers. Significant conceptual and methodological gaps will be identified. Evidence gathered through this environmental scan can support decision-making and assist health care organizations to respond, adapt, and build on potential challenges and opportunities.

## Appendix

Multimedia Appendix 1 Peer-review report by the Québec-Flanders Bilateral Research Cooperation Program - The Research Foundation Flanders - Fonds de recherche du Québec (FRQ).

## Abbreviations

MCI: mild cognitive impairment
NCD: neurocognitive disorders

## Data Availability

All data from this environmental scan will be made available through an online repository.
