# Supplementary material for: Existing eHealth Solutions for Older Adults Living With Neurocognitive Disorders (Mild and Major) or Dementia and Their Informal Caregivers: Protocol for an Environmental Scan
Source: JMIR Res Protoc. 2022 Nov 4;11(11):e41015. doi: 10.2196/41015 (PMC9675011; doi:10.2196/41015)
Supplement: Multimedia Appendix 1 [file resprot_v11i11e41015_app1.pdf]

Québec
Fonds de recherche – Nature et technologies
Fonds de recherche – Santé
Fonds de recherche – Société et culture

fwo Research Foundation Flanders
Opening new horizons

2017 Québec-Flanders Bilateral Research Cooperation Program

PRE-REPORTER – Scientific evaluation form

Information about the project to evaluate

	Québec	Flanders
Principal Investigator	Marie-Pierre Gagnon	Ronald Buyl
University :	Universite laval	VUB Techtransfer
Project title :	*Promoting informed and shared decisions about eHealth solutions for older people and their informal caregivers*	

Application Number :<br><br>Applicant_QC :<br><br>Applicant_FR :	Pre-reporter:

EVALUATION OF THE APPLICATION

Criteria 1: Quality of the project

Criteria variables:

- Scientific quality
- Methodology
- Originality and innovative nature of the project

Comments (there is no limit; the box adjusts to the length of the text):

Scientific quality

The proposal is well-written and describes the development and testing of a decision support system for older people with cognitive impairment to select eHealth solutions. A participatory approach is proposed to engage with several target groups: people at risk of cognitive impairment, those with mild cognitive impairment (MCI), people with dementia, family caregivers, health and social care providers. Based on the needs and preferences of these groups a broad range of solutions classified as eHealth will be categorised and offered on a website. The reviewers all felt that the scientific quality is good but all point out limitations and weaknesses with the overall concept and delivery. One reviewer also notes that the overall idea is not new as similar systems have been attempted before in Europe but also points out that developing an underlying model of decision making for eHealth solutons would be a valuable addition to the field.

Methodology

The proposed work is split into four phases which largely follow a sequential model with an interview study over the first 15 months, environmental scan in months 13-18, development of the decision tool in months 19-33 and evaluation in months 30-36. All three reviewers highlight limitations and weaknesses in the proposed design and methodology. First relates to the cognitive ability and capacity to consent of the intended participants. The project proposes to include “those with mild to severe dementia” but the section on ethics states that only adults with the capacity to give consent will be approached. They also state that no groups who are considered vulnerable will be approached to participate. Furthermore, no details are provided about how the severity of cognitive impairment or capacity to consent of potential participants will be assessed or who on the team has the training to do this.

A second concern relates to the time allocated to each phase of the project and the start and end point of each phase. This is particularly so in creating the decision support tool which should commence much earlier, ideally at the start of the project. This process needs to be iterative and co-created with the intended end-users over many months. Also, several innovative interactive approaches to working with older adults to identify their needs and preferences have been developed in recent years that provide more information and insight than interviews as participants typically find it difficult to articulate detailed needs and wishes when asked but can demonstrate these through interaction with existing or prototype devices.

A third methodological concern relates to the evaluation phase which comes late in the project but few details are provided as to how this will be undertaken. This may reflect lack of expertise or experience in this aspect of the proposed work. Given that this aspect of the work is so important it would beneficial to adopt an explicit iterative approach with discrete develop and test cycles with end-users to ensure usabiity, an approach which has been successfully used by other groups to develop technologies in partnership with people who have dementia.

Further concerns relate to differences in health systems, markets/provision/support, legal and statutory regulations and definitions of medical products and assistive technology. Additionally, one reviewer questions the inclusion of two small groups (n=10) of health and social care providers in the proposal. It is not clear what level or role in their organisation these participants will be or what the purpose of including them is as the project objectives all relate to older adults at risk of or already living with cognitive impairment and informal caregivers.

Originality

The reviewers agree that the project is using cutting edge approaches and addressing a current real-world need given both the expected rise in numbers of older adults living with cognitive impairments and the rapid increase in (unregulated and largely non-evidenced) consumer products targeting this population. One reviewer also highlighted the need for the team to understand and acknowledge existing devices, applications and web-based resources as a starting point rather than presenting the study as a blank slate.

Application Number :<br>Applicant_QC :<br>Applicant_FR :	Pre-reporter:

Criteria 2: Impact of the project

Criteria variables:

- Purposefulness of the project, dissemination, knowledge transfer and visibility
- Impact on the training of highly qualified personal

Comments (there is no limit; the box adjusts to the length of the text):

Purposefulness, dissemination, knowledge transfer and visibility

Project has a clear goal and objectives and if successful could have a high impact on older adults living with cognitive impairment.

Dissemination is enhanced by the partners membership of various interprofessional networks and their intention to access other existing networks such as AGE-WELL and the Canadian Frailty Network. The involvement of health and social care professionals and organisations should promote visibility of the project and needs to be complemented by presentations and publications to professional and practice audiences.

Knowledge transfer to partner organisations is laid out but less is said about the means of informing older adults at risk of or living with cognitive impairment about the decision support tool.

One issue raised by the reviewers is the need to maintain a web-based resource and keep this up to date as new solutions are released, i.e. beyond the end of the project.

HQP

No specific details are provided about the nature of the training the HQP will receive.

**Application Number :**<br>**Applicant_QC :**<br>**Applicant_FR :**	**Pre-reporter:**

Criteria 3: Quality of the team

Criteria variables:

- International scientific level of the research groups
- Complementarity/synergy between researchers regarding the selected topic and articulation of the interdisciplinarity (see Criteria 2.5)
- Added value of the international collaboration
- Balanced distribution between Flemish and Québec researchers

Comments (there is no limit; the box adjusts to the length of the text):

Research groups

The reviewers all agree that the overall quality of the team is good with a mix of newer and more experienced investigators. Members in each site have worked together but this is the first time the whole team is coming together. Collectively they have an excellent track record of attracting funding and working as principal investigators, co-investigators and collaborators.

Complementarity

The team covers psychology, health and mechanical engineering but expertise in HCI, usability and interface design are noted as valuable additions. One reviewer recommends the formation of an advisory board of external experts, including knowledge users and people representing a more ‘applied’ perspective.

Added value

The differences in health and education systems are highlighted but the specific added value of the two teams is not directly described.

Balance between teams

Balance of the work and responsibilities is reasonable.

Application Number :<br><br>Applicant_QC :<br><br>Applicant_FR :	Pre-reporter:

Criteria 4: Organization and management

Criteria variables:

- Quality of the planning
- Feasibility of the project
- Efficient use of existing resources
- Necessity for the budgeted resources

Comments (there is no limit; the box adjusts to the length of the text):

Planning

The project is laid out over four phases and with the proposed sample sizes, completing the work should be possible. However, there is a risk that the evaluation (late in the project) will reveal the decision support system is not usable or liked by the intended end-users. This can be addressed through starting the decisions support system devleopment early, trialing a limited model and developing some use cases. A second concern relates to the dependencies between pahses 1 and 2 whereby the proposed categories may not produce a system that can successfully categorise the technologies.

Feasibility

The project as proposed is feasible but the risks created by dependencies need to acknowledged.

Resources

On reviewer raises issues with two proposed budget items: First is funding for a librarian starting in year 2, which the reviewer feels is too late and should commence at the start of the project (year 1). Second is the budgeting of 60 hours for a programmer which the reviewer suggests it too little.

Budget

Overall the budhget seems reasonable and includes the cost of teleconferencing which is presumably to ensure regular team meetings. Funds for purchasing existing eHaelth solutions does not appear in the budget (unless the evaluation is not going to acutally involve the physical devices and apps). The cost of the annual meeting seems large for room and refreshments (C$15000 for three meetings) unless this includes team travel.

**Application Number :**<br><br>**Applicant_QC :**<br><br>**Applicant_FR :**	**Pre-reporter:**

Interdisciplinarity/-sectoriality of the project

Pertinence of the project interdisciplinarity and intersectorality: Applicants are expected to propose coherent interdisciplinary/-sectoral research projects covering research domains falling under at least two of the three following Québec Research Funds:

- FRQNT: Natural Sciences, mathematics and engineering
- FRQS: Health Sciences
- FRQSC: Social and Human Sciences, Arts and Letters

More information on the domains covered by each Québec Research Fund can be consulted on their respective webpages (see weblinks above).

Degree of interdisciplinarity/-sectoriality:

NOT covering domains of at least 2 of the 3 Québec Funds AT ALL				FULLY covering domains of at least 2 of the 3 Québec Funds
1	2	3	**4**	5

Comments (there is no limit; the box adjusts to the length of the text):

The project falls within the Health Sciences and Social and Huamn Sciences themes. The team covers a range of domains but may benefit from an HCI or user-centred design member.

**Application Number :**<br>**Applicant_QC :**<br>**Applicant_FR :**	**Pre-reporter:**

GLOBAL RATING

Compared it with all the projects you could have previously reviewed or been in contact with, you consider this project:

General comments and other remarks (there is no limit; the box adjusts to the length of the text):

Overall the project is interesting and well-written with some limitations relating to the timing of the different stages, the lack of detail about the evaluation, and issues relating to consent and capacity.

**Application Number :**	**G0D8518N**	**Referee :**
**Applicant_QC :**	**Marie-Pierre Gagnon**	
**Applicant_FR :**	**Ronald Buyl**	

1. EVALUATION OF THE APPLICATION

1.1 Criteria 1: Quality of the project

Criteria variables:

- Scientific quality
- Methodology
- Originality and innovative nature of the project

Comments (there is no limit; the box adjusts to the length of the text):

Scientific quality: This is a well written proposal that aims to develop, using participatory methods, an electronic decision support tool for people with dementia and their carers. The proposal draws on current evidence and academic literature to make a case for their study. The scientific quality overall is good.

Methodology: Overall the methodology is well described and they highlight the expertise of the consortium and investigators. The project has 4 interrelated work packages and a participatory approach is used throughout. The methodology for each work package is appropriate and the proposal gives good detail as to how each work package will be run. They have also considered ethical issues that may arise.

There are a few details that I would have liked to see within the methodology. The project will be recruiting people with MCI, dementia and carers, and they will be using existing established relationships with partners to recruit these. However, they do not describe how they will make an assessment on whether a person has MCI or dementia, and to what severity. They have stated that only adults with capacity to give informed consent will be involved in the research, but how will capacity be judged? Although they do state that as a team they have the expertise to carry out research with this population of participants.

The qualitative work package aims to interview 60 participants and two focus groups will also be carried out with health and social care providers. This sample spans Quebec and Flanders. Two focus groups (one in each site) feels like an add on, and if they feel speaking to health and social care professionals is integral to the development of their tool then I would expect this number to be higher. What is the purpose of these focus groups?

The proposal spans different countries and they argue that this means it is a 'transcontinental collaboration' that will be able to interrogate the development of such a tool within different healthcare and education systems. I agree that the different health systems will impact on the availability, design and use of technologies, however they do not pick this up specifically later in the proposal. How will this be analyzed and integrated into the development stage?

The pilot study is a small sample, particularly as it spans the two sites, but this is not unusual.

The investigators state that the evaluation strategy will be developed during the course of the project, however I would have expected to have some details as to evaluative methods within the proposal.

Originality and innovative nature:

Designing, developing and implementing a decision aid tool in this area of research is original to my knowledge. Embracing a more participatory approach is also a good approach and the way in which research is now moving. My only concern, or question, is how does this decision tool get updated when new and more innovative ehealth interventions are developed and put on the market?

1.2 Criteria 2: Impact of the project

Criteria variables:

- Purposefulness of the project, dissemination, knowledge transfer and visibility
- Impact on the training of highly qualified personal

Comments (there is no limit; the box adjusts to the length of the text):

The proposal and team have a good plan in place for dissemination and knowledge transfer. They are asking for over C$16000 for open access publications, I personally think this is high, but it ensures that combined with conference money requests, the research will be made visible within the academic community.

The project sits within a good number of networks which will ensure that the project has impact

1.3 Criteria 3: Quality of the team

Criteria variables:

- International scientific level of the research groups
- Complementarity/synergy between researchers regarding the selected topic and articulation of the interdisciplinarity (see Criteria 2.5)
- Added value of the international collaboration
- Balanced distribution between Flemish and Québec researchers

Comments (there is no limit; the box adjusts to the length of the text):

The quality of the team is good and each investigator has an excellent track record in attracting funding and publications. The team is multi-disciplinary and covers the expertise that I would expect to see in such a proposal. They highlight that there is a mix of established and newer researchers which is always good to see in a proposal as it means that we are capacity building. They acknowledge that these teams have not worked together previously and this could be a risk to the project. However individually the teams are good.

The added value of the international collaboration is not explicitly drawn out, although they do touch on how the different health and education systems is important. But as I stated earlier I would have expected to see more description as to what this adds , how they manage this difference within the de sign, development and implementation of the decision aid etc.

The balance seems to be equitable between Flemish and Quebec researchers.

1.4 Criteria 4 : Organization and management

Criteria variables:

Application Number :	G0D8518N	Referee :
Applicant_QC :	Marie-Pierre Gagnon	
Applicant_FR :	Ronald Buyl	

- o Quality of the planning
- o Feasibility of the project
- o Efficient use of existing resources
- o Necessity for the budgeted resources

Comments (there is no limit; the box adjusts to the length of the text):

The timescales presented seem feasible and the sample isn't too large, which would be a risk. I think there is scope to increase some aspects (for example the number of focus groups if it has a clear aim and adds to the project). The budget seems fine, although I did note earlier that publication costs were high (but in the total costs this is only a small percentage).

1.5 Interdisciplinarity/-sectoriality of the project

Pertinence of the project interdisciplinarity and intersectorality: Applicants are expected to propose coherent interdisciplinary/-sectoral research projects covering research domains falling under at least two of the three following Québec Research Funds in which the added value of the collaboration, and the complementarity between partners, is clearly shown:

- o FRQNT: Natural Sciences, mathematics and engineering
- o FRQS: Health Sciences
- o FRQSC: Social and Human Sciences, Arts and Letters

More information on the domains covered by each Québec Research Fund can be consulted on their respective webpages (see weblinks above).

Please indicate one of the underneath boxes in order to score the degree of interdisciplinarity/-sectoriality of the project and motivate your score in the comment box.

NOT covering domains of at least 2 of the 3 Québec Funds AT ALL				FULLY covering domains of at least 2 of the 3 Québec Funds
1	2	3	**4 X**	5

Comments (there is no limit; the box adjusts to the length of the text):

As stated before the team cover the disciplines that I would expect to see within a proposal of this nature. Individually they bring different skills and expertise. I was pleased to see that for the qualitative component of the study stipulate that a researcher with a social science background is needed.

**Application Number :**	**G0D8518N**	**Referee :**
**Applicant_QC :**	**Marie-Pierre Gagnon**	
**Applicant_FR :**	**Ronald Buyl**	

2. GLOBAL RATING

Compared it with all the projects you could have previously reviewed or been in contact with, you consider this project:

General comments and other remarks (there is no limit; the box adjusts to the length of the text):

This is a well written proposal that draws on current evidence to make a good justification of why this study should be done. The team have had excellent success in attracting funding and publishing high quality publications. There were some details within methods that I felt were missing (particularly around defining and categorising the sample, assessing capacity to consent, and ensuring each data collection phase collects enough data). These are all details that can be though about when delivering the study. The investigators might consider the researchers to get trained specifically with assessing capacity etc.

**Application Number :**	**G0D8518N**	
**Applicant_QC :**	**Marie-Pierre Gagnon**	**Referee :**
**Applicant_FR :**	**Ronald Buyl**	

1. EVALUATION OF THE APPLICATION

1.1 Criteria 1: Quality of the project

Criteria variables:

- Scientific quality
- Methodology
- Originality and innovative nature of the project

Comments (there is no limit; the box adjusts to the length of the text):

First, the consortium present itself as very experienced one – both with respect to the overall issue at stake here as well as with respect to the project coordination. The consortium is interdisciplinary and in addition relies on an interprofessional network. The idea of developing an electronic decision support tool is not new however. There have been several attempts of the last couple of years and there exist some solutions, which might count for a solution tool. Some of them promise advice but only offer a catalogue of solutions; others have focused on the group of elderly with e. g. dementia but only make recommendations in very general terms. But they definitely lack underlying models. They are web-based or app-based.

Therefore, second, it is a must for the project at hands that the most recent developments in that area are integrated in the overall project activities. The project must not start with the understanding that there is nothing valuable in the market so far. The project team however is right in two observations, first, most solutions in that field (even those developed in the EU in the context e.g. AAL) are two small scaled and in the end only few people can profit from services and advice. The cooperation of Quebec and Flanders might constitute a kind of micro-ecosystem (if the team succeeds to keep the interprofessional actors on board) but with the underlying model they would offer transfer knowledge to other similar attempts. Second, language is a crucial issue with people aging and diagnosed with dementia. Having a service available in the respective (everyday) languages would be a valuable service. However, it is not only language, it is culture, the way of how we do or understand thinks which needs to be looked at.

Third, in most EU projects, a cross-European perspective should guarantee that many different conditions, e.g. legal conditions, are reflected on. Also this project - as all the others – needs to decide how to meet these differences. In the end it will be used somewhere by someone.

Fourth, from a methodological point of view the project is state of the art; it has a user-centered design which is a must condition and highly welcomed. The recruitment of participants however is crucial and needs to be looked at more closely – in particularly under the light of legal and ethical concerns. The project team comments in the ethical section that they do not see the problem that persons might not be able to give informed consent nor do they consider working with vulnerable individuals or groups, or that they would work with patients. This needs to be clarified; elderly people and people with MCI or mild dementia signs need under certain circumstances be understood as vulnerable, if related to a hospital or caring facility are from an insurance perspective understood patients. The project team presents itself very experienced, and I do not doubt their sensitivity in that field. But this might has to be looked at in more detail also under the premises of the international attempt (e.g. legal and ethical requirements in the different states). The same is true for the issue of personal data. With respect to dementia almost everything is sensitive personal data. (See phase one of the project).

Fifth, the work packages make sense in their outline. However, WP 1 is far too long; 2 starts too late and could be shorter. WP 3 is crucial and if the project wants to successfully have to offer an electronic tool at the end there is the need to shift WP 3 and WP 4 more into focus. As a general remark, the WPs could

be better integrated with each other. Service Design / Prototyping has much short cycles! I miss a description of the design and functionality of the tool (WP3).

Sixth, more interactions, beyond the planned in dissemination meetings, are necessary. Monthly conference calls are a must to scccessfully work together. Working meetings are very different to dissemination meetings; and one would not want to run the risk that after 12 months the basic decision taken are questioned.

1.2 Criteria 2: Impact of the project

Criteria variables:

- Purposefulness of the project, dissemination, knowledge transfer and visibility
- Impact on the training of highly qualified personal

Comments (there is no limit; the box adjusts to the length of the text):

The project plan foresees regularly dissemination activities both for the scientific community as well as for and through the interprofessional network. That is an overall plus! With that, the project has a very high recognition degree. It can be assumed that the professionals involved will learn the functionality and possibilities of use in the ongoing process rather being presented with a final version.

It is clear that the tool as such will be the best communication tool for the project itself and its attempts. However, the project team needs to think about other communication option and clearly stress the group of audiences they want to serve with their results. A list of potential journals as well as conferences surely could be provided.

Experience with other projects in that field have shown that it is necessary to have the most important stakeholders on board. Therefore, beyond the platform the project team should seek to inform the communities from the beginning on; they need to understand the functionality. Beyond scientific journals there is the need to publish and inform also through specific professional media.

The involved interprofessional stakeholder network however guarantees the spread of information of the project. It has to be orchestrated and should not only take place as suggested in WP 4 only.

1.3 Criteria 3: Quality of the team

Criteria variables:

- International scientific level of the research groups
- Complementarity/synergy between researchers regarding the selected topic and articulation of the interdisciplinarity (see Criteria 2.5)
- Added value of the international collaboration
- Balanced distribution between Flemish and Québec researchers

Application Number :	G0D8518N	Referee :
Applicant_QC :	Marie-Pierre Gagnon	
Applicant_FR :	Ronald Buyl	

Comments (there is no limit; the box adjusts to the length of the text):

I already stressed earlier that the research team as well as the interprofessional network involved can be considered the very strong pillar of that project. (please see comments above)

If at all there is one component missing. I would build up an advisory board consisting of experts from the field but not only from the two regional areas. It is always tricky if you become to much concerned with the regionalization of something that is an overall issue. Furthermore, there is maybe a need to included some more “applied” perspectives.

The project is clearly supervised and responsibility between the partners are clearly distributed.

1.4 Criteria 4 : Organization and management

Criteria variables:

- o Quality of the planning
- o Feasibility of the project
- o Efficient use of existing resources
- o Necessity for the budgeted resources

Comments (there is no limit; the box adjusts to the length of the text):

See above

Staff type	Technicians - parttime	2019	C$ 5000.00
Motivation	Librarian for help with systematic review		
Name	Unknown Unknown		
Academic degree			
Current employer			
Staff type	Technicians - parttime	2020	C$ 4500.00
Motivation	1 Programmer for the development of the decision aid (60 hours at $75/hour)		
Name	Unknown Unknown		
Academic degree			
Current employer			

Application Number :	G0D8518N	Referee :
Applicant_QC :	Marie-Pierre Gagnon	
Applicant_FR :	Ronald Buyl	

This does not really make sense for me; first 2019 for a literature review is too late. The project needs to work on the state of the art right from the beginning, so why not start in 2018. Second, 60 hours for a programmer is not enough. I would be very cautious in that field. From the data provided I cannot see who takes responsibility and how the development/design of the tool takes place. But I suppose the planned in PhD students is taken responsibility for the model.

With respect to consumables to consider: equipment and software solutions interviews (data collection and analysis); dissemination events, documents/prints beyond scientific publications.

1.5 Interdisciplinarity/-sectoriality of the project

Pertinence of the project interdisciplinarity and intersectorality: Applicants are expected to propose coherent interdisciplinary/-sectoral research projects covering research domains falling under at least two of the three following Québec Research Funds in which the added value of the collaboration, and the complementarity between partners, is clearly shown:

oFRQNT: Natural Sciences, mathematics and engineering
xFRQS: Health Sciences
xFRQSC: Social and Human Sciences, Arts and Letters

More information on the domains covered by each Québec Research Fund can be consulted on their respective webpages (see weblinks above).

Please indicate one of the underneath boxes in order to score the degree of interdisciplinarity/-sectoriality of the project and motivate your score in the comment box.

NOT covering domains of at least 2 of the 3 Québec Funds AT ALL				FULLY covering domains of at least 2 of the 3 Québec Funds
1	2	3	4	x

Comments (there is no limit; the box adjusts to the length of the text):

2. GLOBAL RATING

Compared it with all the projects you could have previously reviewed or been in contact with, you consider this project:

General comments and other remarks (there is no limit; the box adjusts to the length of the text):

Very good but not excellent

But I consider the task and the proposed solution independently from its regional focus as a very important contribution. While the researchers attempts might be more towards pure research, is the prospect from an applied research perspective to have a functioning tool based on a model - that supports people with their very specific needs – very interesting.

**Application Number :** **G0D8518N**<br>**Applicant_QC :** **Marie-Pierre Gagnon**<br>**Applicant_FR :** **Ronald Buyl**	**Referee :**

1. EVALUATION OF THE APPLICATION

1.1 Criteria 1: Quality of the project

Criteria variables:

- o Scientific quality
- o Methodology
- o Originality and innovative nature of the project

Comments (there is no limit; the box adjusts to the length of the text):

In a nutshell, the project aims at developing a decision support system (DSS) to help elderly with cognitive problems and their informal caregivers find suitable IT-based solutions for getting support in their daily lives so as to maintain and improve their quality of life and autonomy, esp. regarding their ability to stay at home instead of having to move to a care unit.

As the first steps towards developing such a DSS the project plans to conduct empirical research to

- find out what the needs, wishes and priorities of potential users of such IT-based support systems ("eHealth solutions") are, and
- identify the systems on the market and to create a characterization of each system.

The DSS then basically takes up the results of this empirical research to map wishes and needs of a specific user to the descriptions of the available systems and to come up with suggestions suited to a particular need.

The methodology for the empirical research (WP1 and WP2) is sound, well-thought-out and nicely elaborated. However, there seems to be a weakness as well. In phase 1 interviews will be conducted and the transcripts be analyzed using thematic data analysis. A central result of this analysis is a category system which reflects the various needs and expectations found in the interviews and focus groups. While this is a sound procedure it might prove difficult to use these categories to properly characterize the eHealth solutions on the market, which will be scanned in the second phase of the project. It might turn out that the category system needs to be extended and adapted to properly describe the eHealth solutions, possibly requiring follow-up interviews of potential end users for clarification. In other words, crucial for the success of the project will be the category system which not only adequately describes the needs of potential users but also the existing solutions. The description of the methodology does not address such interactions between phases 1 and 2.

The project plans to use the Decision Box Approach to develop the DSS. This is a state-of-the-art choice as far as clinicians are the target audience. However, aiming at potential end users of eHealth solutions this might not be the best choice and should therefore be at least be reflected more critically. Moreover, the textual descriptions of the eHealth solutions in the Decision Box Approach might be difficult to understand by the end users who are neither eHealth nor IT experts. In this respect the project could adopt a more visionary approach and think about alternative ways to communicate with potential users and help them understand which kinds of eHealth solutions might suit their needs. For example, the project could work with use cases – a quite common approach in requirements analysis. With use cases the potential benefits and drawbacks of an eHealth system can be communicated much better since they frame the relevant information in a way the potential end user can immediately understand. Other, more innovative approaches to conduct a decision dialog with an end user could use graphical means (or even augmented reality), e.g. to visualize how they would be embedded in a user's apartment (sensor placements, etc.).

The objectives of the project are inherently interdisciplinary and require a combination of expertise from various fields, such as Active Assisted Living (AAL), eHealth, geriatrics, information & communication technologies, human-computer interaction, empirical research.

1.2 Criteria 2: Impact of the project

Criteria variables:

- Purposefulness of the project, dissemination, knowledge transfer and visibility
- Impact on the training of highly qualified personal

Comments (there is no limit; the box adjusts to the length of the text):

The project addresses a very important topic. Today there exists a multitude of powerful eHealth and AAL solutions on the market but it is nearly impossible to identify those that fit a specific need and to make an informed decision on selecting the proper solution for a specific person and situation. This not only holds true for the target users of such a system, i.e. elderly and their informal caregivers, but also for care specialists, nurses, and physicians. Providing a DSS to help make informed decisions would therefore be of great value. The involved research groups are prominent players in the area of AAL and eHealth and therefore in a good position to disseminate the resulting system and to work with patient organisations as well as decision makers to make the system widely available. According to the work plan the last 6 months of the project are dedicated to these dissemination activities.

While the eHealth solutions considered by the DSS are necessarily country-specific the underlying logic is not and can be re-used in other countries. Furthermore, the DSS would be a great tool for training professional caregivers to help them better understand how to assess the needs of elderly as well as show them which solutions exist and inform them about their strengths, weaknesses and requirements.

An important aspect somewhat neglected by the consortium is the life cycle management of the DSS. Not only will new solutions enter the future market but with new technologies becoming practical there will also be new types of systems available. This might have an impact on the categories underlying the DSS as well as the decision rules. Furthermore, besides the technical aspects of maintaining and updating the DSS there is also the issue of who will be in charge of this in an organisational sense. These issues can certainly be solved but would need to be addressed more explicitly.

1.3 Criteria 3: Quality of the team

Criteria variables:

- International scientific level of the research groups
- Complementarity/synergy between researchers regarding the selected topic and articulation of the interdisciplinarity (see Criteria 2.5)
- Added value of the international collaboration
- Balanced distribution between Flemish and Québec researchers

**Application Number :**	**G0D8518N**	**Referee :**
**Applicant_QC :**	**Marie-Pierre Gagnon**	
**Applicant_FR :**	**Ronald Buyl**	

Comments (there is no limit; the box adjusts to the length of the text):

The research groups involved in the proposal have a high scientific standing and are internationally visible. They have considerable experience with research projects and provide the expertise needed for the project. Additional expertise in HCI (human-computer interaction), usability, and user interface design would help the project to address the issue of how to design the DSS so that it is best suited to the task of communicating the characteristics of possible eHealth solutions to the target users, i.e. elderly and their informal caregivers.

The partners have a considerable degree of overlapping expertise (both covering the area of eHealth) but also complement each other: the Quebec group has more expertise and experience with daily care issues while the Flanders group covers more the medical aspects.

The international collaboration creates an added-value, which is especially important for the kind of project being proposed since it requires a broad coverage of needs, wishes and decision criteria elderly have concerning eHealth solutions on the one hand, and a broad coverage of existing eHealth solutions on the other hand. It would also be interesting to find out if and how different the markets in Canada and Belgium are. Cultural differences will give a broader perspective and help develop a DSS that can be more easily transferred into the settings of other countries.

The involvement and financial support of both groups is well balanced and adequate.

1.4 Criteria 4 : Organization and management

Criteria variables:

- Quality of the planning
- Feasibility of the project
- Efficient use of existing resources
- Necessity for the budgeted resources

Comments (there is no limit; the box adjusts to the length of the text):

The project plan is straightforward and reflects the three project phases. The planning provides for sufficient involvement of patient organisations. Based on the methodology, which is well elaborated, the project is perfectly feasible but has the risk that pilot testing might show that the DSS is difficult to use and does not give the intended support for selecting eHealth solutions. Since pilot testing comes in the last third of the project there would not be much opportunity to account for any deficiencies with an adapted design. To reduce the risk, preliminary testing with a limited decision model and a few focused use cases should be done as early as possible in the project.

A similar argument holds for resolving possible dependencies between project phases 1 and 2 . The category system developed in phase 1 might turn out to be insufficient to properly characterize the eHealth solutions found in phase 2, making a mapping between needs and system characteristics difficult. A possible second iteration of phase 1 should therefore be accounted for.

The planned resources are adequate. The work plan sets up an efficient use of these resources.

Application Number :	G0D8518N	Referee :
Applicant_QC :	Marie-Pierre Gagnon	
Applicant_FR :	Ronald Buyl	

1.5 Interdisciplinarity/-sectoriality of the project

Pertinence of the project interdisciplinarity and intersectorality: Applicants are expected to propose coherent interdisciplinary/-sectoral research projects covering research domains falling under at least two of the three following Québec Research Funds in which the added value of the collaboration, and the complementarity between partners, is clearly shown:

- FRQNT: Natural Sciences, mathematics and engineering
- FRQS: Health Sciences
- FRQSC: Social and Human Sciences, Arts and Letters

More information on the domains covered by each Québec Research Fund can be consulted on their respective webpages (see weblinks above).

Please indicate one of the underneath boxes in order to score the degree of interdisciplinarity/-sectoriality of the project and motivate your score in the comment box.

NOT covering domains of at least 2 of the 3 Québec Funds AT ALL				FULLY covering domains of at least 2 of the 3 Québec Funds
1	2	3	4	5

Comments (there is no limit; the box adjusts to the length of the text):

Score: 4

The project clearly falls within FRQS (Health Sciences). Additionally, it addresses aspects of FRQSC, especially the aspects

- "Communication Contexts" (under "Media, Communication and Information"): The DSS needs to provide information relative to the context of the user who seeks advice from the system

- Education, Knowledge and Skills: The DSS can serve as a means for training of professional caregivers

- "Family" and "Social Services" (under "The Development and Functioning of Individuals, Communities and Social Living"): Supporting the care of elderly within their own homes has lots of implications for families, which quite often take a huge part of the burden (of informal care).

These additional aspects from FRQSC are clearly there but mostly only implicitly addressed by the proposal and not fully elaborated. Despite this lack of elaboration in the proposal I would give a score of 4 since the aspects from FRQSC are definitely in the scope of the project.

2. Global Rating

Compared it with all the projects you could have previously reviewed or been in contact with, you consider this project:

**Application Number :**	**G0D8518N**	
**Applicant_QC :**	**Marie-Pierre Gagnon**	**Referee :**
**Applicant_FR :**	**Ronald Buyl**	

General comments and other remarks (there is no limit; the box adjusts to the length of the text):

Here again the main weaknesses:

- not sufficiently addressing usability and more advanced modes of communication with end users of the DSS

- not addressing life cycle management of the DSS, esp. the lack of considering extensibility

- the project plan follows too strict the classical waterfall model and should allow more feedback loops and iteration between the project phases
